# *Jẹ̀díjẹ̀dí,* free sugar consumption and early childhood caries experience in Ile-Ife, Nigeria: a cultural dimension to dental caries risk

**DOI:** 10.3389/froh.2025.1608125

**Published:** 2025-09-17

**Authors:** Moréniké Oluwátóyìn Foláyan, Roberto Ariel Abeldaño Zuñiga, Omolola Titilayo Alade, Oluwabunmi Tope Bernard, Olaniyi Arowolo, Taofeek Kolawole Aliyu, Olusegun Stephen Titus, Simin Z. Mohebbi, Mohammad R. Khami

**Affiliations:** 1 Research Center for Caries Prevention, Dentistry Research Institute, Tehran University of Medical Sciences, Tehran, Iran; 2 Department of Child Dental Health, Obafemi Awolowo University, Ile Ife, Nigeria; 3 Postgraduate Department, University of Sierra Sur, Oaxaca, Mexico; 4 Centre for Social Data Science, Faculty of Social Sciences, University of Helsinki, Helsinki, Finland; 5 Department of Preventive and Community Dentistry, Faculty of Dentistry, College of Health Sciences, Obafemi Awolowo University, Ile Ife, Nigeria; 6 Mary Heersink School of Global Health and Social Medicine, Faculty of Health Sciences, McMaster University, Hamilton, ON, Canada; 7 Department of Languages and Cultures, Ghent University, Ghent, Belgium; 8 Department of Sociology and Anthropology, Obafemi Awolowo University, Ile-Ife, Nigeria; 9 Department of American Studies, Konstanz University, Konstanz, Germany; 10 Department of Music, Obafemi Awolowo University, Ile Ife, Nigeria; 11 Community Oral Health Department, School of Dentistry, Tehran University of Medical Sciences, Tehran, Iran

**Keywords:** dental caries, ethnomedicine, traditional, culture, Nigeria, risk factors, dietary sucrose, preschool

## Abstract

**Background:**

*Jẹ̀díjẹ̀dí* is a Yoruba ethnomedical gastrointestinal phenomenon associated with reduced refined carbohydrate consumption. This study assessed the associations between maternal belief in “*jẹ̀díjẹ̀dí*” and age of sugar introduction into children's diets, daily frequency of refined carbohydrate consumption between meals, and early childhood caries (ECC) experience in Ile-Ife, Nigeria.

**Methods:**

This study involved a secondary analysis of cross-sectional data collected from 878 mother-child dyads residing in Ile-Ife Central Local Government Area between December 2024 and January 2025. Participants were selected through a multi-stage random sampling process. Data were collected using structured, interviewer-administered questionnaires as well as clinical dental examinations that used the decayed, missing, and filled teeth (dmft) index. Three separate multivariable logistic regression models were employed to assess the association between maternal belief in *jẹ̀díjẹ̀dí* and three oral health outcomes (age of introduction of sugar into diet, frequency of consumption of refined carbohydrate between meals daily, and ECC experience). These models adjusted for covariates (socioeconomic status as a contextual factor; the child's age, sex, use of fluoride toothpaste, and toothbrushing frequency as child-level factors; and the mother's age and knowledge of caries prevention as mother-level factors).

**Results:**

Among the 878 children included in the study, 538 (61.3%) had been introduced to refined carbohydrates before their first birthday, 202 (23.0%) consumed refined carbohydrates more than three times per day between meals, 713 (81.2%) expressed belief in *jẹ̀díjẹ̀dí*, while 70 (8.0%) children have ECC. Maternal belief in *jẹ̀díjẹ̀dí* was associated with non-significant trends suggesting a possible delay in sugar introduction (adjusted odds ratio [AOR]: 1.119; 95% confidence interval [CI]: 0.776–1.614; *p* = 0.547) and a lower frequency of refined carbohydrate consumption (AOR: 1.412; 95% CI: 0.942–2.115; *p* = 0.095). There was no significant association observed between belief in *jẹ̀díjẹ̀dí* and the presence of ECC (AOR: 1.002; 95% CI: 0.516–1.947; *p* = 0.995).

**Conclusion:**

While maternal belief in *jẹ̀díjẹ̀dí* was not significantly associated with the oral health outcomes, it demonstrated a tendency toward protective dietary practices. Future studies should explore leveraging *jẹ̀díjẹ̀dí* within culturally tailored ECC prevention programs.

## Introduction

Culture profoundly shapes oral health beliefs, behaviours, and practices, influencing dietary choices, hygiene routines, and perceptions of disease (1, 2). Traditional practices frequently observed across diverse communities include the frequent consumption of sugary snacks and sweetened beverages, the use of non-fluoridated oral hygiene agents (e.g., charcoal or herbal pastes) instead of evidence-based fluoride toothpaste, prolonged breastfeeding or bottle-feeding (especially at night), and the cultural normalization of dental caries as an unavoidable part of childhood (3–6). These practices are well-established risk factors for early childhood caries (ECC), a disease that causes cavities in the teeth in children younger than 72 months of age (3, 5, 6). Furthermore, historical or cultural mistrust of dental professionals can contribute to delayed care-seeking, exacerbating oral health issues (7).

Conversely, some cultural beliefs may inadvertently offer protection against ECC. An example relevant to dental caries is *jẹ̀díjẹ̀dí*, a culturally constructed illness concept within Yoruba ethnomedicine. *Jẹ̀díjẹ̀dí* is primarily perceived and diagnosed as a gastrointestinal and systemic condition (e.g., diarrhoea, bloating, perianal irritation, general weakness, and concerns about future sexual dysfunction, particularly in males) believed to arise from excessive consumption of sugar, fatty foods, and carbonated drinks (8–10). Crucially, the belief in this illness concept shapes healthcare-seeking and dietary habits, leading to sugar restriction as a traditional treatment (10, 11). Maternal belief in *jẹ̀díjẹ̀dí* has been associated with reduced sugar consumption in children (8, 9). Similar culturally rooted concepts regulating sugar intake exist in other parts of Eastern Nigeria and West Africa, suggesting broader cultural relevance for preventive strategies (9–11).

This study is theoretically anchored in the PEN-3 model, a framework designed to examine health behaviours through the lens of culture, relationships, and community contexts (12). The PEN-3 model comprises three domains: (1) Cultural Identity (Person, Extended Family, Neighborhood), (2) Relationships and Expectations (Perceptions, Enablers, Nurturers), and (3) Cultural Empowerment (Positive, Existential, Negative). Applied here, the model positions *jẹ̀díjẹ̀dí* as a culturally embedded belief system within the Cultural Identity domain, influencing maternal perceptions (Relationships and Expectations) about sugar's health impacts. By examining how this belief empowers caregivers to restrict sugar intake (Cultural Empowerment), the study explores whether *jẹ̀díjẹ̀dí* serves as a culturally resonant protective factor against ECC risk behaviours.

Despite growing evidence that *jẹ̀díjẹ̀dí* can influence dietary behaviour and sugar intake patterns among caregivers (8, 9), its direct association with measurable oral health outcomes remains poorly understood. It is not yet clear whether maternal belief in *jẹ̀díjẹ̀dí* delays the introduction of sugar into infants' diets, reduces the frequency of refined carbohydrate consumption between meals, or lowers the risk or prevalence of ECC. Moreover, the potential to harness this culturally resonant belief in the design of targeted, preventive oral health programs has not been adequately explored. This gap limits the development of effective, culturally grounded interventions for ECC in populations where such beliefs significantly shape caregiving practices and child health behaviours (13).

This study aims to examine the association between maternal belief in *jẹ̀díjẹ̀dí* and three key outcomes: the age at which sugar is first introduced into children's diets, the daily frequency of refined carbohydrate consumption between meals, and the experience of ECC among children aged 6 to 71 months in Ile-Ife Central Local Government Area of Osun State, Nigeria. By investigating these relationships, the study seeks to evaluate whether the culturally embedded concept of *jẹ̀díjẹ̀dí* could inform the development of culturally sensitive public health strategies for ECC prevention in similar West African contexts.

## Methods

### Ethical considerations

Ethical approval for the study was secured from the Tehran University of Medical Sciences, Tehran, Iran (IR.TUMS.DENTISTRY.REC.1402.023) and the Institute of Public Health Research Ethics Committee of the Obafemi Awolowo University, Ile-Ife, Nigeria (IPH/OAU/12/2742). Written informed consent was obtained from mothers to allow their children to participate. Confidentiality was strictly maintained, with no participant identifiers such as names or residential addresses included in the dataset. Participants were not provided with any form of compensation for their involvement.

### Study design

This was a secondary analysis of a cross-sectional study that recruited 1411 mother-child pairs in Ife Central Local Government Area, Osun State, Nigeria, between December 2024 and January 2025 to determine the risk factors for ECC.

### Study population and study location

The study targeted children aged 6 to 71 months residing with their primary caregivers in Ile-Ife, Nigeria. The sole eligibility criterion was the provision of written informed consent by a primary caregiver. No other exclusion criteria were applied, and all children present at home during the study period who met this criterion were eligible to participate. This approach aimed to ensure broad representation of the study population.

### Sample size

The required sample size for the primary study was determined using the Cochran formula, based on an estimated 4.3% prevalence of ECC (14). With a 5% margin of error and a 95% confidence level, the sample size was 64 children. However, to ensure adequate power for multivariable analysis at the 4.3% prevalence, 1,628 children needed to be screened to identify 70 cases of ECC (70 ÷ 0.043). The primary study recruited 1,411 mother-child dyads. In the final analytic sample (*n* = 878), 70 ECC cases (8.0%) were identified, meeting the requirement for robust regression analysis.

### Sampling procedure

A multi-stage random sampling method was employed. From the 700 enumeration areas in the 2006 National Population Census, 70 (10%) were selected for the study participants' recruitment. The 70 sites were the same sites where prior ECC-related studies had been conducted in Ile-Ife in 2014 and 2020 (14, 15). Within each enumeration area, every other household on each street was screened to identify eligible mother-child dyads. Only one mother-child dyad was enrolled per household, even if multiple eligible children resided there. This ensured statistical independence of observations and minimized intra-household clustering effects in dietary and behavioural practices (16).

Eligible households that declined participation after explanation of the study objectives were skipped, and the next eligible household on the same street was approached as replacements to maintain the sampling interval. Recruitment continued until all designated enumeration areas were covered, with only one dyad per household participating. The recruitment process continued for six weeks, with further recruitment discontinued once the eligible enumeration areas were covered.

In areas with irregular or non-linear layouts, field teams applied a flexible definition of “street”, treating any continuous pedestrian-accessible pathway (including footpaths and alleys) as a valid sampling route. Households along these routes were systematically enumerated. In sparsely populated areas, sampling was extended to include adjacent paths when the initial street did not yield enough eligible households. Vacant or non-residential buildings were skipped, and the next occupied household was selected, with all skipped structures documented to preserve sampling transparency. In locations where gated houses made access to the compound challenging, the house was skipped, but the random selection of houses was maintained. The community engagement before study commencement enhanced access for the field workers to households at the enumeration areas.

### Data collection

Data were collected using interviewer-administered questionnaires and dental examinations. The same instrument was used for studies conducted in 2014 and 2020 (14, 15), and it captured a broad range of variables. These variables included the socio-demographic characteristics of the child, such as socioeconomic status, as well as oral health behaviours like the child's toothbrushing frequency and use of fluoridated toothpaste (17), and maternal knowledge of caries prevention (18). The questionnaire also addressed child feeding practices, focusing on the age of sugar introduction and the frequency of refined carbohydrate consumption between meals, along with cultural practices, particularly maternal belief in *jẹ̀díjẹ̀dí.* This belief was assessed using the question, “Do you take steps to protect your child from *jẹ̀díjẹ̀dí?*” with binary “Yes” or “No” response options. Before the main data collection, the instrument was pilot-tested with 20 mother-child dyads who were not included in the final sample. The pilot, conducted by the 20 trained data collectors, aimed to evaluate public comprehension and the overall flow of the questionnaire. It also helped identify challenges with skipping logic and phrasing due to the questionnaire's online format. However, no modifications were made to the instrument following the pilot.

The data collectors participated in a three-day program led by the principal investigators. The first day was an online training introducing all participants to the study protocols. The next two days were physical training that took participants through the study questionnaire, and training on ethical research conduct, including obtaining informed consent and maintaining participant confidentiality, and provided instruction on standardized questionnaire administration. Participants also learnt about effective communication to enhance responses by participants. Practical sessions, such as role-playing, were included to prepare the team for real-world challenges in field interviews.

In addition, clinical data collection protocols were reviewed to support dental examination procedures. Nine calibrated dentists conducted caries assessments using the dmft index, with the intra- and inter-examiner reliability scores computed using Fleiss' Kappa. Intra-examiner reliability averaged 0.86 (range: 0.78–0.92) across duplicate assessments of 10 children repeated two days after the initial assessment. The initial inter-examiner reliability score was 0.62 (*p* = 0.349), prompting additional training on identifying non-cavitated lesions to refine diagnostic accuracy in alignment with the lead consultant.

All dental examinations were carried out under good illumination. Standard cross-infection control protocols were implemented: examiners wore disposable gloves, masks, and protective eyewear for each child examined. Sterilized mouth mirrors were used for the examinations. Gauze and other single-use items were collected in disposable trash bags and discarded at the hospital incinerator at the end of the day.

### Study variables

Interviews with mothers or guardians collected data on socio-demographics, oral health behaviours of both mother and child, and child feeding practices.

#### Dependent variables

##### Early childhood caries experience

ECC was defined as cavitated or non-cavitated caries in primary teeth of children aged ≤5 years (19). The dmft index was used to quantify ECC, counting decayed, filled, or missing teeth due to caries (20). Clinical examinations were performed in a well-lit room (natural and/or artificial light) within the participants' house, with the child seated upright on a chair or a stable caregiver's lap. Teeth were cleaned of debris using sterile gauze where necessary. Examinations were conducted using sterilized plane mouth mirrors and portable LED headlamps. Parents provided explanations for any unexplained tooth loss, and ECC status was categorized as present (dmft > 0) or absent (dmft = 0) for analysis.

##### Frequency of refined carbohydrate consumption between meals daily

Participants were asked to report the frequency of their consumption of refined carbohydrates as snacks or beverages between their main meals daily. The response options were structured as follows: “About 3 times a day or more” was coded as 1, “About twice a day” as 2, “About once a day” as 3, “Occasionally; not every day” as 4, and “Rarely or never eat between meals” as 5. In addition, a code of 99 was assigned for instances where no response was provided.

##### Age of introduction of sugar into the meal of child

Mothers/caregivers were asked to indicate when sugar was introduced into their child's diet by responding to: *At what age was sugar included in [CHILD'S NAME]'s diet?* Options ranged from “*At birth*” to “*After 12 months*”, with additional choices for “*Cannot remember*” and “*No Response*”. Responses were dichotomized into sugar introduced “before one year of age” and “at or after one year of age”*,* creating a binary variable: Sugar introduction at or after 1 year of age (yes/no) (21).

#### Confounding variables

##### Socioeconomic status

Socioeconomic status was assessed using an adapted version of the index developed by Olusanya et al. (22), which had been previously applied in studies within the same setting (23). This index integrates multiple factors, combining the mother's level of education with the father's educational attainment and occupation.

For this study, data were collected on the parents' educational levels and professions. The mother's education was categorized into three groups: no formal education, Quranic and primary school education (score 2); secondary school education (score 1); and tertiary education (score 0). The father's occupation was classified into three levels: civil servants or skilled professionals with tertiary education (score 1); civil servants or skilled professionals with secondary education (score 2); and unskilled workers, unemployed individuals, students, or civil servants/skilled professionals with primary or Quranic education (score 3).

The socioeconomic class of each child was determined by summing the mother's education score and the father's occupation score. Children were then assigned to one of five social classes: Class I (upper class), Class II (upper middle class), Class III (middle class), Class IV (lower middle class), and Class V (lower class). In cases where only one parent was alive or available, the classification was based on the available parent's data. If both parents were deceased or unavailable, the child's primary caregiver (e.g., grandparent, aunt, or legal guardian) was used as a proxy, applying the same scoring criteria. For statistical analysis, the five social classes were collapsed into three categories: high (classes I and II), middle (class III), and low (classes IV and V).

##### Age

The age was established as the child's age on their last birthday.

##### Sex

Sex was determined as male or female.

##### Maternal oral health knowledge

Parental oral health knowledge was assessed using an instrument that had been used in a prior study (17). This study focused on data collected from mothers, who responded to eight statements on caries diagnosis and prevention using a five-point Likert scale ranging from “strongly agree” to “do not know”. Statements covered topics such as fluoride use, sugar consumption, sealants, and the importance of regular dental visits.

Responses of “strongly agree” and “agree” were considered correct, with scores assigned from 5 (strongly agree) to 1 (do not know). Non-responses were scored as 1. Each participant could obtain a minimum score of 8 and a maximum of 40. The mean score of the group was used to determine the cut-off point. Mothers scoring 15 and above were classified as having good oral health knowledge, while those scoring 14 or below were categorized as having poor oral health knowledge.

##### Child's toothbrushing frequency

The mothers or guardians were asked how often their child's teeth were brushed, with response options ranging from “Irregularly or never” to “twice a day or more” and “No response”. Acceptable levels, defined as “twice a day or more”. Acceptable level of behaviour was coded “1” and others as “0”.

##### Children's use of fluoridated toothpaste

In addition, questions were asked on the regularity of using fluoridated toothpaste when brushing their teeth. They could respond with one of the following options: “Always”, “Quite often”, “Seldom”, or “Not at all”. The acceptable level of use of fluoridated toothpaste was “always/quite often. Acceptable level of behaviour was coded “1” and others as “0”.

#### Independent variable

##### Maternal cultural belief in jẹ̀díjẹ̀dí

To assess whether caregivers actively take steps to protect their children from *jẹ̀díjẹ̀dí*, participants were asked a direct question regarding their preventive measures. They were specifically asked whether they were taking any actions to safeguard their child from *jẹ̀díjẹ̀dí*, with response options categorized as “Yes” or “No”. All “No Responses” were treated as “No”.

### Data analysis

The dataset comprised both categorical and numerical variables. For variables with less than 5% missing data, a complete case analysis was performed under the assumption that the missing values were completely random (MCAR). For variables with 5%–15% missingness, diagnostic tests (Little's MCAR test and examination of missingness patterns using cross-tabulations and logistic regression) were conducted to evaluate the missingness mechanism. Where these diagnostics suggested missingness was ignorable (MCAR or MAR), numerical data were imputed using the median, while categorical data were imputed using the mode to preserve data integrity (24). Variables with more than 15% missingness were carefully assessed, using the same diagnostics. If missingness was determined to be non-random (likely MNAR) or strongly associated with key variables, sensitivity analyses were conducted to evaluate its potential impact on the findings (e.g., comparing complete case analysis to analyses using different plausible imputation values or excluding the variable) (24). Analyses using imputed data were compared with complete case analyses to ensure consistency in results.

In addition, preliminary screening for multicollinearity was conducted using Pearson correlation coefficients for continuous variables (child's age, maternal age). For categorical variables (socioeconomic status, sex), variance inflation factors (VIFs) were computed post-model specification. Variables with a correlation exceeding 0.7 were removed. All VIF values in the final logistic regression models were <5 (mean VIF range: 1.2–1.8), well below the threshold of 10, indicating no severe multicollinearity. Standard errors were examined for instability, with no anomalies detected. Models were re-run, excluding variables with moderate correlations (maternal age and socioeconomic status). Results for the primary exposure (*jẹ̀díjẹ̀dí* belief) remained non-significant (AOR range: 1.10–1.42; *p* > 0.05), confirming robustness. Hierarchical regression (contextual → child-level → mother-level variables) ensured coefficient stability during covariate introduction.

Three separate multivariable logistic regression models were constructed to assess the associations between the independent variable (maternal cultural belief in *jẹ̀díjẹ̀dí*) and the three oral health outcomes (age of sugar introduction, frequency of refined carbohydrate consumption, and ECC experience). The analysis was conducted in a stepwise manner, with each level of variables introduced sequentially, followed by a final model that includes all variables. The three levels of variables were: contextual (socioeconomic status of the child), child-related (age, sex, frequency of use of fluoridated toothpaste, twice daily tooth brushing or more), and mother-related (maternal knowledge of caries prevention, and age of mother). Each table provides odds ratios (OR), adjusted odds ratios (AOR), 95% confidence intervals (CI), and *p*-values for each variable. Statistical significance was set at *p* < 0.05. Model fit was evaluated using the Hosmer-Lemeshow test (all *p* > 0.05), and Nagelkerke's *R*^2^ values (range: 0.08–0.22) indicated acceptable explanatory power. Residual analyses revealed no influential outliers. The analysis was conducted using SPSS v. 25.

## Results

Table 1 shows the data of the 878 mother-child dyads with enough information for the data analysis to ensure the validity of multivariable regression models. Of these, 538 (61.3%) children were introduced to sugar before the age of one year, 202 (23.0%) children consumed sugar more than three times a day, and 70 children had ECC. In addition, 713 (81.2%) of mothers believed in *jẹ̀díjẹ̀dí.*

**Table 1:** Frequencies of responses to questions related to maternal knowledge [*N* = 878].

Variables	Number	Percentage
Maternal cultural belief in *jẹ̀díjẹ̀dí*		
Yes	713	81.2
No	165	18.8
Age of introduction of sugar into the diet		
≤1 year	538	61.3
>1 year	340	38.7
Frequency of refined carbohydrate consumption between meals daily		
<3 times a day	676	77.0
≥3 times a day	202	23.0
ECC experience		
Yes	70	8.0
No	808	92.0

The mean age of the children was 2.7 years (±1.5 SD). Of the 878 children, 421 (47.9%) were males and 457 (52.1%) were females. Most children (73.0%, *n* = 641) used fluoridated toothpaste, though only 102 (11.6%) brushed their teeth ≥2 times/day. Mothers had a mean age of 30.9 years (±5.8 SD) and a mean knowledge score of 19.1 (±9.8 SD) for caries prevention.

Table 2 shows that at the contextual level, the SES of the child was a significant predictor of ECC. In the unadjusted model, children from middle and high socioeconomic backgrounds had lower odds of developing ECC compared to those from low SES backgrounds. However, only children from high SES had statistically significantly lower odds of having developed ECC (OR: 0.472; *p* = 0.027).

**Table 2:** Logistic regression analysis for the association between maternal cultural belief and early childhood caries [*N* = 838].

Variables	Total (*n*, %)	1st level: contextual				2nd level: child's variables				3rd level: mother's variables				All variables in the final model			
		OR	95% Confidence interval			OR	95% Confidence interval			OR	95% Confidence interval			AOR	95% Confidence interval		
			LL	UL	*p* value		LL	UL	*p* value		LL	UL	*p* value		LL	UL	*p* value
Socioeconomic status (Child) Low Middle High	200 (22.8) 354 (40.3) 324 (36.9)	1.000 0.847 0.472	0.474 0.243 –	1.513 0.918 –	0.575 0.027 –	– – –	– – –	– – –	– – –	– – –	– – –	– – –	– – –	1.000 0.817 0.410	0.450 0.200 –	1.483 0.839 –	0.506 0.015 –
Age (Child)	2.7 (SD 1.5)	–	–	–	–	1.529	1.266	1.846	<0.001	–	–	–	–	1.592	1.307	1.941	<0.001
Sex (Child) Male Female	421 (47.9) 457 (52.1)	– –	– –	– –	– –	1.041 1.000	0.634 –	1.709 –	0.874 –	– –	– –	– –	– –	1.048 1.000	0.636 –	1.727 –	0.855 –
Use of fluoridated toothpaste (Child) Yes No	641 (73.0) 237 (27.0)	– –	– –	– –	– –	1.000 0.906	0.494 –	1.659 –	0.748 –	– –	– –	– –	– –	1.000 0.872	0.461 –	1.651 –	0.674 –
Twice daily tooth brushing or more (Child) Yes No	102 (11.6) 776 (88.4)	– –	– –	– –	– –	1.000 0.718	0.350 –	1.474 –	0.367 –	– –	– –	– –	– –	1.000 0.665	0.312 –	1.416 –	0.290 –
Age (mother)	30.9 (SD 5.8)	–	–	–	–	–	–	–	–	0.991	0.950	1.034	0.588	0.973	0.931	1.018	0.238
Maternal knowledge of caries prevention	19.1 (9.8)	–	–	–	–	–	–	–	–	0.997	0.973	1.022	0.832	1.007	0.979	1.036	0.612
Maternal cultural belief in *jẹ̀díjẹ̀dí* Yes No	758 (80.0) 193 (20.0)	– –	– –	– –	– –	– –	– –	– –	– –	1.138 1.000	0.596 –	2.174 –	0.695 –	1.002 1.000	0.516 –	1.947 –	0.995 –

At the child level, the age of the child was a strong predictor, with older children having higher odds of ECC: for every year increase in age, the odds of ECC increased by 52% (*p* < 0.001) in the unadjusted model. The sex of the child was not significantly associated with ECC. The use of fluoridated toothpaste and the frequency of tooth brushing were not significantly associated with ECC.

At the mother level, neither the mother's age nor her knowledge of caries prevention was significantly associated with ECC. Maternal cultural belief in *jẹ̀díjẹ̀dí* also showed no significant association with ECC (OR = 1.138; *p* = 0.695) in the unadjusted model.

In the final adjusted model, maternal cultural belief in *jẹ̀díjẹ̀dí* showed no significant association with ECC (AOR:1.002; 95% CI: 0.516–1.947; *p* = 0.995).

Table 3 shows that no statistically significant associations exist between SES and the age of introduction of sugar into the diet of the child. However, the age of the child was significantly associated with the timing of sugar introduction: the odds of introducing sugar at one year or later increased by 17% for each additional year of age (OR: 1.170; *p* = 0.001) in the unadjusted model. Children who used fluoridated toothpaste had significantly higher odds of introducing sugar at one year or later compared to those who did not (OR: 0.592; *p* = 0.002). In addition, children who brushed their teeth twice daily or more had significantly higher odds of introducing sugar at one year or later (OR: 0.588; *p* = 0.014), and mothers with higher knowledge of caries prevention had slightly higher odds of introducing sugar at one year or later (OR: 1.016; *p* = 0.023). The sex of the child, age of the mother, and maternal belief in *jẹ̀díjẹ̀dí* did not show a significant association with the timing of sugar introduction.

**Table 3:** Logistic regression analysis for the association between maternal cultural belief and the age of introduction of sugar into the diet of the child (one year or after) [*N* = 838].

Variables	Total (*n*, %)	1st level: contextual				2nd level: child's variables				3rd level: mother's variables				All variables in the final model			
		OR	95% Confidence interval			OR	95% Confidence interval			OR	95% Confidence interval			AOR	95% Confidence interval		
			LL	UL	*p* value		LL	UL	*p* value		LL	UL	*p* value		LL	UL	*p* value
Socioeconomic status (Child) Low Middle High	222 (23.2) 379 (39.6) 350 (37.2)	1.000 1.203 1.187	0.840 0.823 –	1.723 1.710 –	0.314 0.359 –	– – –	– – –	– – –	– – –	– – –	– – –	– – –	– – –	1.000 1.162 1.030	0.803 0.695 –	1.681 1.526 –	0.427 0.882 –
Age (Child)	2.7 (SD 1.5)	–	–	–	–	1.170	1.063	1.288	0.001	–	–	–	–	1.197	1.084	1.322	<0.001
Sex (Child) Male Female	457 (48.1) 494 (51.9)	– –	– –	– –	– –	0.953 1.000	0.722 –	1.258 –	0.734 –	– –	– –	– –	– –	0.960 1.000	0.727 –	1.269 –	0.776 –
Use of fluoridated toothpaste (Child) Yes No	686 (72.3) 265 (27.7)	– –	– –	– –	– –	1.000 0.592	0.424 –	0.826 –	0.002 –	– –	– –	– –	– –	1.000 0.628	0.444 –	0.888 –	0.009 –
Twice daily tooth brushing or more (Child) Yes No	108 (11.3) 843 (88.7)	– –	– –	– –	– –	1.000 0.588	0.385 –	0.897 –	0.014 –	– –	– –	– –	– –	1.000 0.627	0.405 –	0.969 –	0.036 –
Age (mother)	30.9 (SD 6.3)	–	–	–	–	–	–	–	–	0.988	0.965	1.012	0.336	0.979	0.955	1.003	0.090
Maternal knowledge of caries prevention	19.1 (9.8)	–	–	–	–	–	–	–	–	1.016	1.002	1.030	0.023	1.009	0.994	1.025	0.233
Maternal cultural belief in *jẹ̀díjẹ̀dí* Yes No	758 (80.0) 193 (20.0)	– –	– –	– –	– –	– –	– –	– –	– –	1.256 1.000	0.880 –	1.793 –	0.209 –	1.119 1.000	0.776 –	1.614 –	0.547 –

In the final adjusted model, maternal cultural belief in *jẹ̀díjẹ̀dí* showed no significant association with the age of introduction of sugar into the diet of the children (AOR:1.119; 95% CI: 0.776–1.614; *p* = 0.547) although mothers with cultural beliefs in *jẹ̀díjẹ̀dí* had higher odds of introducing sugar into the diet of their children at one year or later.

Table 4 indicates that there was no significant association between the SES, age, and sex of the child, daily use of fluoridated toothpaste, maternal age, and maternal cultural belief in ***jẹ̀díjẹ̀dí*** and the frequency of consumption of refined carbohydrate in-between-meals less than three times a day. However, maternal knowledge of caries prevention was strongly associated with reduced frequency of consumption of refined carbohydrates in-between meals, less than three times a day (OR: 0.952; *p* < 0.001).

**Table 4:** Logistic regression analysis for the association between maternal cultural belief and the frequency of refined carbohydrates consumption in-between-meals daily (less than three times a day) [*N* = 838].

Variables	Total (*n*, %)	1st level: contextual				2nd level: child's variables				3rd level: mother's variables				All variables in the final model			
		OR	95% Confidence interval			OR	95% Confidence interval			OR	95% Confidence interval			AOR	95% Confidence interval		
			LL	UL	*p* value		LL	UL	*p* value		LL	UL	*p* value		LL	UL	*p* value
Socioeconomic status (Child) Low Middle High	222 (23.2) 379 (39.6) 350 (37.2)	1.000 0.767 0.830	0.503 0.539 –	1.171 1.279 –	0.219 0.398 –	– – –	– – –	– – –	– – –	– – –	– – –	– – –	– – –	1.000 0.855 1.288	0.552 0.804 –	1.324 2.063 –	0.483 0.293 –
Age (Child)	2.7 (SD 1.5)	–	–	–	–	0.944	0.847	1.052	0.300	–	–	–	–	0.913	0.813	1.024	0.119
Sex (Child) Male Female	457 (48.1) 494 (51.9)	– –	– –	– –	– –	0.959 1.000	0.699 –	1.314 –	0.793 –	– –	– –	– –	– –	0.911 1.000	0.658 –	1.262 –	0.575 –
Use of fluoridated toothpaste (Child) Yes No	686 (72.3) 265 (27.7)	– –	– –	– –	– –	1.000 1.218	0.837 –	1.770 –	0.303 –	– –	– –	– –	– –	1.000 0.916	0.609 –	1.376 –	0.671 –
Twice daily tooth brushing or more (Child) Yes No	108 (11.3) 843 (88.7)	– –	– –	– –	– –	1.000 0.783	0.466 –	1.318 –	0.358 –	– –	– –	– –	– –	1.000 0.537	0.311 –	0.927 –	0.026 –
Age (mother)	30.9 (SD 6.3)	–	–	–	–	–	–	–	–	0.977	0.950	1.005	0.104	0.980	0.952	1.009	0.172
Maternal knowledge of caries prevention	19.1 (9.8)	–	–	–	–	–	–	–	–	0.952	0.936	0.967	<0.001	0.940	0.923	0.958	<0.001
Maternal cultural belief in *jẹ̀díjẹ̀dí* Yes No	758 (80.0) 193 (20.0)	– –	– –	– –	– –	– –	– –	– –	– –	1.386 1.000	0.832 –	2.063 –	0.107 –	1.412 1.000	0.942 –	2.115 –	0.095 –

In the final adjusted model, maternal cultural belief in *jẹ̀díjẹ̀dí* showed no significant association with the frequency of consumption of refined carbohydrate in-between-meals less than three times a day (AOR:1.412; 95% CI: 0.942–2.115; *p* = 0.095) although mothers with cultural beliefs in *jẹ̀díjẹ̀dí* had higher odds of having children whose frequency of consumption of refined carbohydrate in-between meals was less than three times a day.

## Discussion

This study aimed to assess whether maternal belief in *jẹ̀díjẹ̀dí* influences the age of sugar introduction, frequency of sugar consumption, and ECC prevalence among children aged 6 to 71 months. The findings revealed that maternal belief in *jẹ̀díjẹ̀dí* was not significantly associated with ECC. In addition, maternal belief in *jẹ̀díjẹ̀dí* seemed to increase the odds for delaying the introduction of sugar into the child's diet, and the odds for less frequency of daily consumption of refined carbohydrate in-between meals, though the findings were not statistically significant.

The study has several strengths, including its large sample size and the use of a multi-stage random sampling method, which enhances the generalizability of the findings to the population of Ile-Ife, Nigeria. Its focus on cultural beliefs, such as *jẹ̀díjẹ̀dí*, adds a unique dimension to understanding how traditional practices may influence dietary habits and oral health outcomes. The use of validated tools, such as the dmft index for caries assessment and an adapted socioeconomic status index, further strengthens the study's methodological rigor. However, the cross-sectional design limits the ability to establish causal relationships between maternal belief in *jẹ̀díjẹ̀dí* and ECC. The reliance on self-reported data for sugar consumption, including the timing of sugar introduction and oral hygiene practices, may have introduced recall bias, potentially affecting data accuracy. Additionally, the study's cultural specificity may limit the generalizability of its findings to populations with different belief systems. The modest prevalence of ECC observed—though higher than the 4.3% estimate used for sample size calculation—may have constrained the power to detect subtle associations with cultural beliefs. Other influential factors, such as access to dental care or community-level interventions, were not explored. Despite these limitations, the study provides valuable insights into how culturally informed strategies could support ECC prevention in similar contexts.

The key highlight of the study is the exploration of *jẹ̀díjẹ̀dí* as a cultural phenomenon that may indirectly influence oral health by reducing sugar consumption. Although the study did not find a direct association between *jẹ̀díjẹ̀dí* and ECC, it did reveal that mothers who believed in *jẹ̀díjẹ̀dí* were more likely to delay the introduction of sugar into their children's diets and to reduce the frequency of daily refined carbohydrate consumption. This trend is consistent with earlier ethnographic accounts by Brieger (9) and Omotade et al. (25), who described *jẹ̀díjẹ̀dí* as a culturally driven rationale for restricting sugar and fatty foods in children due to perceived gastrointestinal risks. However, those earlier studies did not quantitatively assess the dietary timing or dental caries outcomes linked to this belief. The divergence between the qualitative alignment and the lack of strong protective association in our findings may reflect shifts in cultural adherence due to modernization or methodological differences, such as the reliance on self-reported recall in our cross-sectional study rather than longitudinal observation. Nonetheless, the observed trend highlights the potential value of culturally grounded beliefs like *jẹ̀díjẹ̀dí* in informing public health interventions aimed at improving childhood nutrition and oral health outcomes. This finding underscores the potential of integrating traditional cultural beliefs into public health strategies to promote healthier dietary practices.

In addition, our finding that maternal belief in *jẹ̀díjẹ̀dí* was associated with a non-significant reduction in between-meal refined carbohydrate consumption offers partial support for the hypothesis proposed by Brimah and Adigun (10), who suggested that cultural beliefs like *jẹ̀díjẹ̀dí* may promote sugar restriction and thus indirectly reduce caries risk. While our results align directionally with this proposition, they fall short of statistical significance. The lack of a significant association in our study may be because the study was not primarily powered to detect this association. Nonetheless, these findings highlight the potential value of exploring culturally grounded practices as a foundation for dietary behaviour change in oral health interventions.

Our analysis also found no significant association between maternal belief in *jẹ̀díjẹ̀dí* and ECC. This finding aligns with existing literature that, while acknowledging the role of Yoruba cultural practices in shaping oral health behaviours, has not specifically examined *jẹ̀díjẹ̀dí* about caries outcomes (4, 26, 27). Previous studies exploring similar ethnomedical beliefs, such as the concept of “jedi-jedi” in Eastern Nigeria, have primarily focused on gastrointestinal conditions rather than oral diseases (11). The relatively low prevalence of ECC in our study population may have further limited the statistical power needed to detect subtle associations.

The study findings are suggestive of the role of indigenous practices in oral health and the need for the integration of cultural practices into oral healthcare systems. The integration of traditional cultural beliefs into public health strategies is increasingly recognized as a valuable approach to improving health outcomes, particularly in communities where traditional practices are deeply rooted (28, 29). This approach can enhance cultural relevance, foster trust, and increase the acceptance of public health interventions (29, 30). However, challenges arise when traditional beliefs or practices, such as the ethnomedical phenomenon known as *jẹ̀díjẹ̀dí*, lack a standardized framework for understanding or management. *Jẹ̀díjẹ̀dí* appears to be a culturally specific health phenomenon characterized by a constellation of symptoms, with interpretations and treatments varying widely, even within the same cultural context (9). This variability poses significant challenges for integrating *jẹ̀díjẹ̀dí* within a broader dental caries prevention program, as cultural sensitivity needs to be balanced with scientific rigor (29).

The PEN-3 model (12) offers a valuable framework for interpreting the findings. Within the Cultural Identity domain, the widespread belief in *jẹ̀díjẹ̀dí* among Yoruba mothers reflects a deeply rooted cultural perspective. However, its lack of significant association with lower ECC prevalence suggests that cultural identity alone is insufficient to drive behavioural change, especially when other factors like socioeconomic status or oral hygiene practices are at play. In the Relationships and Expectations domain, maternal belief that sugar causes *jẹ̀díjẹ̀dí* appeared to encourage reduced sugar intake for children, aligning with protective behaviours. Although these associations were not statistically significant, they indicate a cultural belief shaping behavioural intentions. Still, the impact may be weakened by inconsistent enablers, such as limited support or access to healthier alternatives, and the unmeasured influence of nurturers like elders and traditional healers. The Cultural Empowerment domain recognized *jẹ̀díjẹ̀dí* as a positive, culturally meaningful belief that promotes sugar restriction. However, it also revealed a key limitation: the absence of a direct link to oral health. This gap reduces its effectiveness in promoting broader caries-preventive behaviours such as regular toothbrushing or fluoride use. To enhance its impact, future interventions should integrate *jẹ̀díjẹ̀dí* into oral health messaging, involve community nurturers, and strengthen supportive environments that enable behaviour change for ECC prevention.

Although no previous studies have directly investigated the relationship between *jẹ̀díjẹ̀dí* and ECC, our study helps bridge existing ethnographic insights on the dietary implications of *jẹ̀díjẹ̀dí* with quantitative epidemiological data on dental caries. While the belief is traditionally associated with sugar-related dietary caution, our findings revealed no significant protective association between maternal belief in *jẹ̀díjẹ̀dí* and ECC. This divergence from expectations may reflect a combination of factors, including the possible erosion of traditional beliefs due to modernization, methodological limitations such as the cross-sectional design and recall bias, and the relatively low prevalence of ECC in Nigeria compared to global rates (31), which may reduce the statistical power to detect subtle associations.

To build on these findings, future research should adopt longitudinal designs to explore causal pathways between cultural beliefs, dietary behaviours, and oral health outcomes. Mixed-methods studies could provide deeper context on how *jẹ̀díjẹ̀dí* is interpreted and practiced in contemporary communities. In addition, comparative multi-country research examining analogous cultural beliefs, such as *jedijedi* in Eastern Nigeria or “empacho” in Latin America (32–35), could offer broader insights into how traditional health concepts shape oral health across different settings.

Care must, however, be taken to prevent promoting traditional remedies for dental caries management, as this may further delay access to professional dental care (4, 36). If *jẹ̀díjẹ̀dí* is not perceived as related to oral health, its influence on caries prevention may be negligible, as it does traditional management does not inherently promote a reduction in refined carbohydrate intake for dental caries prevention.

Despite this challenge, the integration of traditional beliefs like *jẹ̀díjẹ̀dí* into public health strategies also presents opportunities (37). These traditional beliefs must be recognized and somehow incorporated into health education efforts, although orthodox health practitioners are often uncomfortable doing this (38). Collaborating with traditional healers to better understand *jẹ̀díjẹ̀dí* and co-develop management strategies could foster trust and improve the cultural relevance of public health programs. Involving communities in research to explore the aetiology, symptoms, and management of *jẹ̀díjẹ̀dí* could help bridge the gap between traditional and biomedical perspectives. This approach could also identify culturally appropriate ways to address oral health within the context of *jẹ̀díjẹ̀dí*. Public health programs could incorporate education about oral health into discussions of *jẹ̀díjẹ̀dí*, helping to align traditional beliefs with preventive health practices. For example, traditional healers could be trained to promote oral hygiene as part of their management of *jẹ̀díjẹ̀dí*.

In conclusion, the study provides valuable insights into the complex interplay between cultural beliefs and oral health outcomes. While *jẹ̀díjẹ̀dí* was not significantly associated with ECC, its association with delayed sugar introduction into the diet of the child and less frequency of consumption of refined carbohydrates daily highlights the potential for culturally sensitive public health interventions. For the ECC prevention program, this might involve tailoring oral health messages to align with local understandings of *jẹ̀díjẹ̀dí*, ensuring that preventive practices are both culturally relevant and scientifically sound. Future research should explore longitudinal designs to better understand the causal relationships between cultural beliefs, dietary practices, and ECC, as well as the role of other environmental and community-level factors in shaping oral health behaviours.

## Data Availability

The raw data supporting the conclusions of this article will be made available by the authors, without undue reservation.
